# An unusual complication of the traditional treatment of a closed fracture – generalized tetanus: a case report

**DOI:** 10.1186/s13256-017-1477-y

**Published:** 2017-10-24

**Authors:** Landry W. Tchuenkam, Emmanuel K. Ndame, Marc L. Guifo, Celestin Danwang, Ginette C. Kalla, Arthur Essomba

**Affiliations:** 1 Faculty of Medicine and Biomedical Sciences, University of Yaounde I, Yaounde, Cameroon; 2 Department of Surgery and Specialties, Faculty of Medicine and Biomedical Sciences, University of Yaounde I, Yaounde University Hospital Center, Yaounde, Cameroon; 3 Department of Pediatric, Faculty of Medicine and Biomedical Sciences, University of Yaounde I, Yaounde University Hospital Center, Yaounde, Cameroon

**Keywords:** Fracture, Forearm, Tetanus, Traditional healer

## Abstract

**Background:**

Tetanus is a severe infectious disease that can lead to death. The clinical manifestations are due to an exotoxin secreted by *Clostridium tetani*, a spore-producing Gram-positive bacillus. The penetration of the germ is made through a skin opening, independently of the size of the wound.

**Case presentation:**

A 13-year-old black African boy of the Bantu ethnic group with unknown tetanus vaccination status presented to our pediatric emergency room for the management of chest and vertebral pains which started a few days after traditional treatment by scarification and herbal and leaf ointment. The treatment was initiated by a traditional healer and indicated for a closed fracture of our patient’s left forearm sustained during a fight. The diagnosis of generalized tetanus was made on the basis of generalized contractures with opisthotonus, trismus, and autonomic nervous system dysfunction. Despite prompt intensive care management, he died a few hours after admission.

**Conclusion:**

This case emphasizes the permanent threat of tetanus in our environment especially after cultural and traditional acts like scarification that in this specific case was for a therapeutic purpose.

## Background

Tetanus is a potentially lethal severe infection due to a neurotropic exotoxin produced by a Gram-positive bacillus, *Clostridium tetani*. It is a non-encapsulated germ able to produce spores that are resistant to heat and the usual disinfectants. Although widely distributed throughout the world, this disease disproportionately affects developing countries [1] due to insufficient immunization coverage. In Cameroon, tetanus vaccine is an integral part of the expanded immunization program, with well-coded vaccine boosters.

The germ penetrates into the body usually through a wound, a bite contaminated by soil, or lack of aseptic measures on surgical material. The microorganism is located at the entrance and the toxin diffuses into the body. The clinical presentation of the disease is variable, ranging from generalized tetanus, localized tetanus, and cephalic tetanus to neonatal tetanus [2]. The diagnosis is essentially clinical while the treatment needs to be considered a matter of emergency [1].

In developed countries, rapid urbanization and introduction of large-scale public health interventions with population education have significantly reduced the incidence of tetanus to sporadic cases [3]. In these regions, its mortality remains very low, less than 0.02 people per 100,000 people per year [4]. In Sub-Saharan Africa, although this rate has decreased by 78.3% since 1990, it remains high with nearly five deaths per 100,000 people [4]. A recent systematic review evaluated the case-fatality rate of tetanus at 43.2% [5] in Africa, compared with 13.2% in developed settings [6]. Sub-optimal care with inadequate access to mechanical ventilation and emergency drugs, and poor access to health care were the main associated factors found in low-income countries [7].

Several studies have demonstrated cases of generalized tetanus where the point of entry remains iatrogenic, following medical care, especially after mastectomy [8], following replantation of an amputated finger [9], after laparotomy [10, 11] and laparoscopic procedure [12], and even following tooth extraction [13].

We report here a case of generalized tetanus whose route of entry remains unconventional: a closed fracture of the forearm; the closed fracture of the forearm had undergone an initial treatment in a traditional manner by scarification and application of a traditional balm.

## Case presentation

This is the case of a 13-year-old black African boy of the Bantu ethnic group, a student, who presented at the pediatric emergency room of Yaounde University Hospital Center with intense chest and vertebral pains, evolving for 48 hours before admission. A week before, in the course of a brawl his left forearm was twisted, resulting in a sharp and permanent pain in his left forearm associated with a functional impotence without any cutaneous lesions. At home, his tutor gave him paracetamol and diclofenac that were administered orally followed by a consultation the next day at a traditional healer. The traditional healer carried out scarifications on our patient’s forearm, consisting of multiple superficial incisions of the skin made by a blade, supplemented by the application on the cutaneous lesions of an ointment composed of herbs, leaves, and earth, which would be likely to contain *Clostridium tetani* spores. Less than 48 hours later, there was an onset of a generalized pain, predominant in our patient’s back and in his sternal region.

The adolescent lives with his aunt in town. There were no elements in favor of a non-accidental injury or child abuse. He had no history of chronic disease; he has never had an operation. The immunization status of the child was unknown to the next of kin.

On general examination on his presentation to our emergency department, he was conscious and ill-looking. His temperature ranged between 36.8 and 38.3 °C, his pulse was 88 beats per minute, his pupils were equal and reactive to light stimulus, and his blood pressure was 105/70 mmHg. He presented a trismus, spinal stiffness, a generalized contracture with abdominal rigidity, and opisthotonus. In addition, there were also spasms triggered by noise, light, and touch during care.

**Fig. 1:**
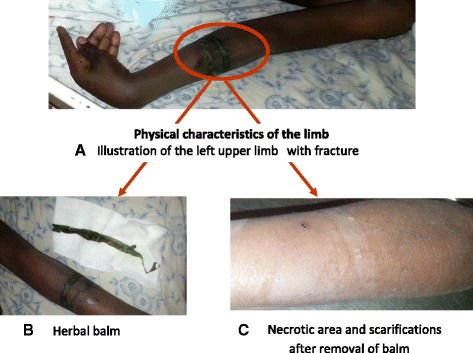
Physical characteristics of the limb. **a** Left upper limb with fracture and area surrounded with balm. **b** Herbal balm. **c** Necrotic area and scarifications are visible after removal of balm

The loco-regional examination of his left upper limb revealed a balm based on herbs and black earth placed under a traditional splint. After removal of the latter, scarifications were visible with areas of cutaneous necrosis (Fig. 1). The rest of the examination was otherwise normal.

**Fig. 2:**
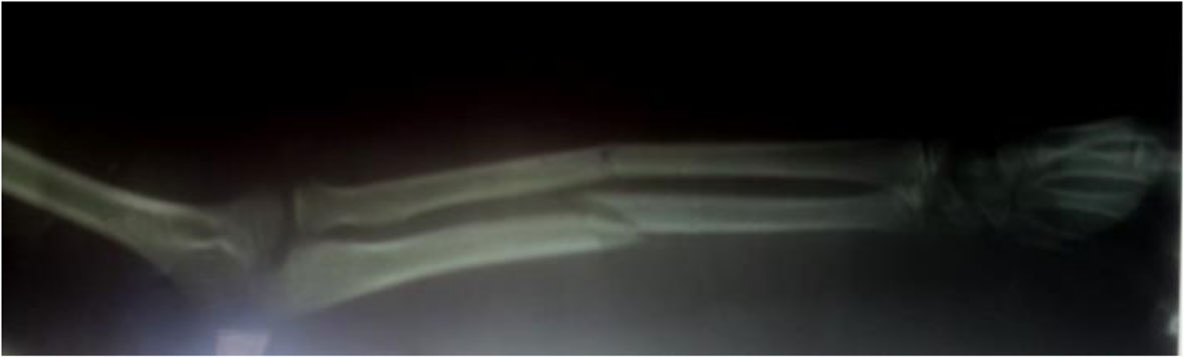
X-ray of left forearm. Fracture of the shaft of the two bones of the forearm classified Orthopaedic Trauma Association/AO 22-A3

An X-ray of his left forearm showed a slightly displaced shaft fracture of the two bones of his forearm classified Orthopaedic Trauma Association (OTA)/AO 22-A3 (Fig. 2). His hematological and blood electrolytes profiles were within normal limits. No biologic test was performed for the detection of tetanus antitoxin antibodies in whole blood due to the non-availability of this test in our setting at the time.

The diagnosis of generalized tetanus complicating a closed fracture of the forearm with subsequent opening was made. Generalized tetanus was thus diagnosed a few hours after our patient’s admission on the basis of the clinical examination in particular, and an obvious point of entry in an individual with unknown vaccination status who clinically presented a trismus, a generalized contracture, as well as paroxysms.

He was subsequently admitted 6 hours later to our intensive care unit. He was placed in solitary confinement in a dimly lit room; a nasogastric tube was placed, and sedation and diazepam myorelaxation were performed. The etiological treatment consisted of an antibiotherapy based on metronidazole that was intravenously, directly, and slowly administered and an immunotherapy with equine anti-tetanus serum. Subsequent disinfection of the point of entry with resection of necrotic tissue was performed to reduce microbial growth and release of toxins.

The evolution was marked by a gradual deterioration in his state of consciousness, dysautonomic manifestations, particularly blood pressure surges, and tachycardia alternating with bradycardia. Moreover, the occurrence of several episodes of tonic–clonic paroxysms required additional doses of muscle relaxant and intubation. He died a few hours later in our intensive care unit, 12 hours after admission to our hospital.

## Discussion

The case of this patient is compatible with a diagnosis of generalized tetanus. The diagnosis was based on a lack of correct vaccination, an iatrogenic entry point, clinically generalized contractures with opisthotonus, a trismus, and autonomic nervous system dysfunction. Generalized tetanus is the most common form of clinical presentation of the disease, accounting for more than 80% of cases [1]. The duration of incubation representing the time between the penetration of the germ and the occurrence of the first symptom varies from 3 to 21 days with a median of 7 days [1]; a short incubation period is an element of poor prognosis. The entry point of the bacterium is made during a skin or mucosal break-in that can be accidental, post-surgical, during medical care, or during ritual practices [14]. The presence of necrotic tissue or foreign bodies makes the environment favorable to the development of the germ. The lethality rate of the pathology varies between 10 and 70% [1]; in Africa this rate is estimated at nearly 45% [5].

The prognostic elements of the disease are related to several severity scores such as the Dakar score [15]. Our patient had a risk of death greater than 50% on the basis of a score of 10: the incubation was less than 7 days (2 points), the invasion was greater than 2 days (1 point), entry point (2 points), the presence of spasms (2 points), a temperature more than 38.4 °C (2 points) and heart rate (1 point). The most recent prediction score [16], the Tetanus Severity Score (TSS), was 10, a result above the threshold value of 8, hence, predictive of death.

Despite the resuscitation measures undertaken, our patient died in the course of an episode of tonic–clonic paroxysm and dysautonomic manifestations.

The sporadic incidence of the cases and the generalization of the vaccination should not make us fail to check the serological status of patients presenting with a wound, even if it is minimal. The “Tetanus Quick Stick” (TQS) test [17] commonly performed in hospitals in developed countries is not available in our health facilities; thus, routine serotherapy is performed when vaccination is not up to date.

In the present case, this was an “iatrogenic” wound performed by a traditional healer. This raises the problem of educating them and the general public about the health risks of some routine traditional practices. The education carried out for the prevention of the transmission of human immunodeficiency virus (HIV)/acquired immune deficiency syndrome (AIDS) and hepatitis B has hidden one of the formidable complications: the contamination of wounds linked to ritual acts by *Clostridium tetani*.

Public health measures, in particular the listing of traditional healers, could allow for continuous education and the suspension of activities of individuals who are a source of danger to the population.

Once diagnosed, the axis of tetanus treatment consists of [18]: the isolation of the patient from various stimuli; wound care through disinfection, cleaning, and removal of necrotic areas; tetanus immunotherapy preferentially of human origin; and antibiotic therapy with metronidazole or penicillin G [19]. Nonspecific measures include control of muscle spasms and autonomic dysfunction, respiratory resuscitation, and nutritional support.

## Conclusions

Tetanus is a potentially life-threatening serious condition. Its eradication requires effective national immunization coverage, the establishment and implementation of post-exposure protocols for traumatic wounds, rigorous asepsis in medical and surgical care, as well as population education and sensitization on risky ritual practices and early hospital referral for a life-saving purpose.

## Abbreviations

AIDS: Acquired immune deficiency syndrome
HIV: Human immunodeficiency virus
OTA: Orthopaedic Trauma Association
TQS: Tetanus Quick Stick
TSS: Tetanus Severity Score
